# Sun protection behaviors, healthcare access, and smoking among melanoma survivors

**DOI:** 10.1007/s11764-024-01727-8

**Published:** 2024-12-09

**Authors:** Jingjing Xie, Maija Kiuru, Brad H. Pollock, Theresa H. M. Keegan

**Affiliations:** 1 Graduate Group of Epidemiology, University of California Davis, One Shields Avenue, Davis, CA 95616 USA; 2 Department of Dermatology, University of California Davis, Sacramento, CA USA; 3 Department of Public Health Sciences, School of Medicine, University of California, Davis, CA USA; 4 Division of Hematology and Oncology, Center for Oncology Hematology Outcomes Research and Training (COHORT), University of California Davis Comprehensive Cancer Center, Sacramento, CA USA; 5 Department of Pathology and Laboratory Medicine, University of California Davis, Sacramento, CA USA

**Keywords:** Melanoma, Survivors, Sun protection, Smoking status, Healthcare access

## Abstract

**Purpose:**

Based on current clinical practice guidelines, melanoma survivors should be advised on the need for sun protection and regular healthcare, as well as smoking cessation, but differences from adults without cancer history are unclear.

**Methods:**

We pooled data from the National Health and Nutrition Examination Survey (2003–2006, 2009–2018), matching 249 melanoma survivors with 498 adults without a cancer history. Adjusted prevalence odds ratios (aPOR) and 95% confidence intervals (CI) were calculated.

**Results:**

One-third of melanoma survivors used multiple sun protection methods, slightly more than adults without cancer. Both groups had < 50% overall sun protection use with long sleeves being the least used. Melanoma survivors who were male (aPOR = 2.91; CI = 1.05–8.06) or had lower education (aPOR = 4.12; CI = 1.65–10.29) were more likely to be current smokers. Older survivors (aPOR = 1.07; CI = 1.03–1.11) were more likely to have health insurance.

**Conclusions:**

Our findings highlight the relatively low use of sun protective practices for melanoma survivors and suggest the need for better counseling on sun protection and smoking cessation, especially for lower education levels and males.

**Implications for Cancer Survivors:**

These findings underscore the importance of enhancing counseling services for sun protection among melanoma survivors and prioritizing smoking cessation support, especially for males and individuals with lower education levels among this population.

**Supplementary Information:**

The online version contains supplementary material available at 10.1007/s11764-024-01727-8.

## Introduction

Cutaneous melanoma is the fifth most common type of cancer in the US, with non-cutaneous melanoma being rarer but more aggressive [1, 2]. Treatment advances offer hope, yet comprehensive survivorship care, including lifestyle changes and surveillance, is vital for improving melanoma survival [3–7].

Cutaneous melanoma survivors are advised to practice sun protection [8–19]. A study reported higher prevalence in melanoma survivors compared to those with no cancer history [20], while others primarily focused on identifying predictors of regular sun protection practices among melanoma survivors alone, without examining differences between this group and the general population [21, 22]. In addition, all melanoma survivors, whether cutaneous or non-cutaneous, are encouraged to have regular follow-up with healthcare providers [8–19]. Adherence to yearly exams is inconsistent and more likely to be missed among the uninsured population, who face barriers to healthcare access and limited resources, potentially leading to worse outcomes [23–26]. Financial struggles, exacerbated by high out-of-pocket costs for care, may discourage survivors from seeking necessary medical services. The existing challenges in accessing healthcare services for melanoma survivors remain unclear.

Recommendation regarding smoking cessation is controversial [8–19], likely because evidence of smoking’s negative impact on cutaneous melanoma prognosis has only emerged recently [27, 28]. The UK National Institute for Health and Care Excellence (NICE) guideline suggests smoking cessation for cutaneous melanoma survivors [12], while US guidelines do not. Research on smoking among melanoma survivors shows mixed findings [27, 29]. One study found similar tobacco use rates between survivors and non-cancer participants [29], while another observed fewer users among melanoma survivors compared to non-melanoma controls [30].

Sun protection and regular healthcare, as well as smoking cessation for survivors, may be crucial elements of survivorship care planning; however, how these practices differ from non-cancer populations remains unclear. Our study used nationally representative US data from the National Health and Nutrition Examination Survey (NHANES) [31] to describe sun protection and regular healthcare, as well as smoking cessation in melanoma survivors compared with those with no cancer history, and, among melanoma survivors, demographic factors associated with access to healthcare and current smoking.

## Methods

### Study population

We obtained data from the National Health and Nutrition Examination Survey (NHANES), a representative sample of civilian, community-dwelling members of the US population using a complex, multistage probability design [31]. Final adult survey response rates for 2003–2016 NHANES were 71% to 84%, and those for 2017–2018 were 52%. We used information from the NHANES (2003–2006 and 2009–2018) surveys, which included 38,855 participants aged ≥ 20 years that completed the Medical Conditions Questionnaire. We excluded 3470 participants with missing cancer history information. We conducted multiple imputations for participants with missing information on education level and poverty income ratio.

Cancer history was determined by the questions, “Have you ever been told by a doctor or other health professional that you had cancer or a malignancy of any kind,” and “What kind of cancer was it.” We classified participants as no cancer history, history of melanoma or history of melanoma and other cancer(s). Due to the limited sample size, we chose to only include melanoma survivors who had a history of another cancer.

For adults aged ≥ 20 years, our study population included 249 patients reporting a history of melanoma (70 also had a history of another cancer). From potential controls without a history of cancer (*n* = 30,253), we matched on age, gender, and race/ethnicity and conducted unconditional logistic regression analysis to obtain the propensity score. We used the propensity score to obtain two matched adults with no history of cancer for each melanoma survivor using greedy algorithm matching, resulting in a matched sample of 498 adults with no history of cancer (Fig. S1).

As only adults aged 20–59 completed the Dermatology Questionnaire, analyses with these data included 66 participants reporting a history of melanoma (9 of whom also had a history of another cancer) and 132 participants without a history of cancer (Fig. S1). Survey weights were applied to account for the complex survey design. Institutional review board approval was not necessary because the data released by the Centers for Disease Control and Prevention were fully deidentified.

### Covariates

Sociodemographic variables included age, gender, race/ethnicity, and education level. As a measure of socioeconomic status, family poverty-to-income ratio (PIR) was calculated by dividing the family income by the poverty guidelines, which is specific to the family size, year assessed, and state of residence, and categorized into low, middle, and high income [32]. For analyses from the Dermatology Questionnaire, we additionally included a categorical variable, sun sensitivity, based on response to sun exposure for 30 min without any sun protection after several months of not going out in the sun: severely sun sensitive (e.g., “severe sunburn with blisters”), mildly sun sensitive (e.g., “turn darker without a sunburn”), and not sun sensitive.

### Outcome variables

Cigarette smoking (smoked at least 100 cigarettes in life) was classified as never, former, and current, as done previously [33]. Measures of healthcare access included whether participants reported being covered by health insurance or some other kind of healthcare plan and had a routine place to go for healthcare when they were sick or needed advice about their health.

Sun protection behaviors were collected in the Dermatology Questionnaire. Respondents were asked three separate questions regarding how often they: (1) stay in the shade; (2) wear a long-sleeved shirt; and (3) use sunscreen when they go outside on a warm sunny day for more than 1 h (always, most of the time, sometimes, rarely or never, refused, don't know, missing). Participants with responses “refused,” “don’t know” or missing values were excluded from the analysis. In NHANES, participants indicated “don’t go out in sun” under question (1) were not asked about question (2) and (3), so any combination in propensity score matching that involved these participants, including melanoma survivors and their matched controls, was not included in the sun protection analyses. Responses were categorized as frequent (always or most of the time), sometimes, or rarely (rarely or never), as done previously [34]. We further defined those reported frequent use of at least 2 of the 3 examined sun-protective methods as having multiple sun protection practices.

### Statistical analysis

Descriptive statistics included assessing distributional characteristics for sun protection behaviors, smoking status, and measures of healthcare access. We used multivariable unconditional logistic regression models to calculate the prevalence odds ratio (POR) for smoking status and healthcare access. The main exposure variable was a history of melanoma, with two adults without cancer matched for each melanoma survivor. In addition, for participants completing the Dermatology Questionnaire, we measured the magnitude of association between history of melanoma with sun protection behaviors, adjusting for covariates. We selected covariates for adjustment a priori based on their known association with the outcomes and/or melanoma history, including age, gender, education level, and PIR; for those completing the Dermatology Questionnaire, we also considered sun sensitivity [34–48].

Among melanoma survivors, we also performed univariable and multivariable logistic regression to measure the association between the outcome variables (healthcare access and smoking status) and demographic characteristics indicated above. Due to the limited sample size (survey data on sun protection behaviors was only available for individuals aged 20–59 years), we were unable to precisely estimate the association between demographic characteristics and sun protection behaviors. To ensure the results’ reliability, we conducted sensitivity analyses by excluding melanoma survivors with another cancer.

## Results

The vast majority of study participants were non-Hispanic White (95.8%), and 64.5% were ≥ 60 years old (Table 1). Compared to adults without a cancer history, melanoma survivors were more likely to be college graduates (72.8% vs. 60.0%) and report a high-income level (54.5% vs. 45.7%). Melanoma survivors reported more frequent use of shade (43.1% vs 25.1%), long sleeves (15.0% vs 2.1%), sunscreen application (49.4% vs 25.9%), and multimodal sun protection (36.2% vs 6.8%) than adults with no cancer history. Additionally, melanoma survivors had a slightly higher prevalence of having health insurance coverage (96.1% vs 94.8%) and a routine place to go for healthcare (95.2% vs 91.8%) than those with no cancer history. For smoking status, melanoma survivors had a high proportion of current smokers, similar to adults with no cancer history (15.6% vs 17.0%).

**Table 1:** Weighted percentages of demographic characteristics, health care access, smoking status, and sun protection behaviors among melanoma survivors and matched adults with no cancer history aged ≥ 20 years, National Health and Nutrition Examination Survey (NHANES)

	Melanoma survivors		Adults with no cancer history	
Among all individuals	*N* = 249	Wt % (SE)*	*N* = 498	Wt % (SE)*
Age				
20–49	32	15.1 (2.8)	64	14.86 (2.2)
50–59	34	20.4 (3.6)	68	20.55 (2.3)
60–79	126	51.5 (4.3)	252	51.6 (2.6)
≥ 80	57	13.00 (1.8)	114	13.03 (1.4)
Gender				
Male	137	51.9 (4.4)	274	50.9 (2.8)
Female	112	48.1 (4.4)	224	49.1 (2.8)
Race/ethnicity				
Hispanic	13	1.4 (0.5)	26	1.6 (0.4)
Non-Hispanic White	223	95.8 (1.1)	446	96.3 (0.7)
Non-Hispanic Black	9	1.1 (0.4)	18	1.2 (0.3)
Other/multiple	4	1.7 (1.0)	8	0.9 (0.4)
Education level				
Less than high school	34	7.9 (1.7)	101	13.74 (1.9)
High school diploma	52	19.3 (3.4)	133	26.29 (2.6)
College degree or higher	163	72.8 (3.7)	264	59.97 (2.7)
Poverty income ratio				
Low-income (≤ 1.3)	42	10.31 (2.0)	129	15.7 (2.0)
Middle-income (> 1.3–3.5)	102	35.22 (3.8)	205	38.6 (2.8)
High-income (> 3.5)	105	54.47 (3.9)	164	45.7 (3.0)
Smoking status				
Never smoker	111	46.9 (4.5)	224	43.3 (2.9)
Former smoker	100	37.5 (4.3)	192	39.8 (2.5)
Current smoker	38	15.6 (2.9)	82	17.00 (2.0)
Covered by health insurance				
No	11	3.9 (1.6)	35	5.2 (1.2)
Yes	238	96.1 (1.6)	463	94.8 (1.2)
Routine place to go for healthcare				
No	11	4.8 (1.7)	39	8.1 (2.0)
Yes	238	95.2 (1.7)	459	91.8 (2.0)
Among individuals aged 20–59 years	*N* = 66	Wt % (SE)*	*N* = 132	Wt % (SE)*
Sun sensitivity				
Not sun sensitive	16	22.4 (6.4)	68	55.5 (5.5)
Mildly sun sensitive	30	44.01 (7.3)	47	35.8 (4.7)
Severely sun sensitive	20	33.5 (7.4)	17	8.7 (2.7)
Staying in the shade^†^				
Rare	13	21.0 (6.1)	39	31.48 (5.7)
Sometimes	22	36.8 (7.6)	54	46.66 (6.1)
Frequent	29	37.7 (6.9)	37	20.21 (4.4)
Don’t go out in the sun	2	4.5 (3.1)	2	1.65 (1.3)
Among sun-exposed individuals aged 20–59 years^#^	*N* = 62^&^	Wt % (SE)*	*N* = 124^&^	Wt % (SE)*
Staying in the shade^†^				
Rare	13	22.8 (6.6)	38	33.3 (5.8)
Sometimes	20	36.4 (7.9)	51	46.9 (6.1)
Frequent	29	40.9 (7.3)	35	19.8 (4.5)
Wearing a long-sleeved shirt^†^				
Rare	39	65.2 (7.3)	73	66.8 (4.9)
Sometimes	11	20.6 (6.2)	36	23.5 (4.2)
Frequent	12	14.2 (5.0)	15	9.6 (3.0)
Using sunscreen on a very sunny day^†^				
Rare	17	30.1 (6.6)	65	43.9 (5.9)
Sometimes	11	19.8 (6.2)	20	22.5 (5.3)
Frequent	34	50.1 (7.4)	39	33.6 (4.6)
Number of frequent sun protection practices^§^				
0	19	38.1 (7.5)	58	50.4 (5.6)
1	18	27.6 (6.5)	45	37.3 (5.1)
≥ 2	25	34.3 (6.7)	21	12.3 (3.6)

*Percentages are weighted according to US census data; Wt % may not add up to 100% because of rounding

^#^Individuals who answered “do not go out in the sun” for the question “stay in the shade?” were defined as self-identified sun-exposed individuals

^&^Participants indicated “don’t go out in sun” were not asked about whether they wear a long-sleeved shirt or use sunscreen (2 melanoma survivors and 2 adults with no cancer history), so any combination in propensity score matching that involved these participants, including melanoma survivors (*n* = 4) and their matched controls (*n* = 8), was not included in the sun protection analyses

^†^Self-reported frequency of sun-protective practice on a warm sunny day

^§^Number of the 3 examined sun-protection techniques (staying in shade, wearing a long-sleeved shirt, using sunscreen) that self-identified sun-exposed individuals reported frequently using on a warm sunny day

**Fig. 1:**
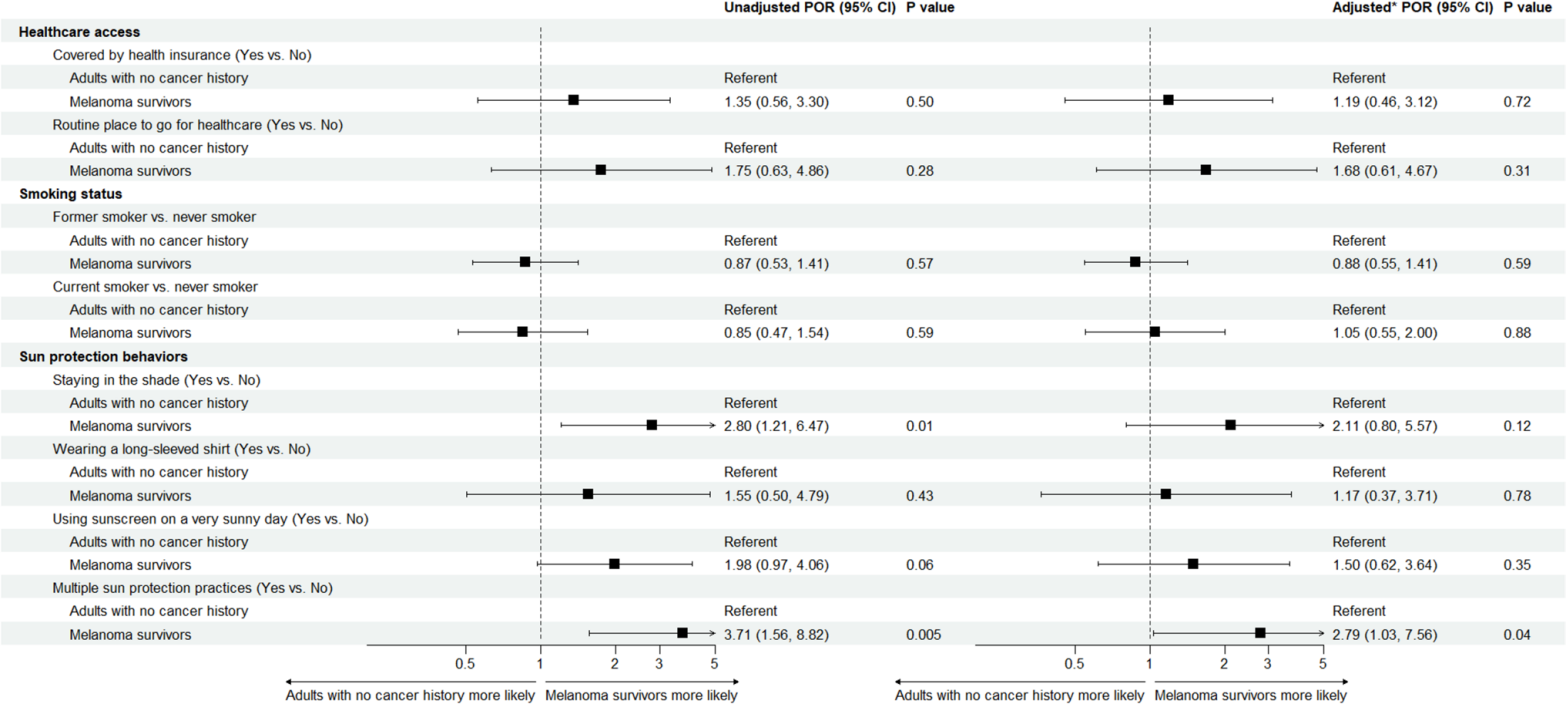
Unadjusted and adjusted* prevalence odds ratio (POR) and 95% confidence interval (CI) of health care access, smoking status, and sun protection behaviors among melanoma survivors and matched adults with no cancer history, NHANES. Those who reported no cancer history were used as the reference group. *Adjusted POR estimates were adjusted for age, gender, race/ethnicity, education level, and poverty income ratio

Melanoma survivors were more likely than those with no cancer history to use multiple sun protection practices (aPOR 2.79; 95% CI 1.03–7.56; *P* = 0.04). Specifically, there was a trend that melanoma survivors used shade (aPOR 2.11; 95% CI 0.80–5.57; *P* = 0.12) and sunscreen more frequently (aPOR 1.50; 95% CI 0.62–3.64; *P* = 0.35), but, these differences were not statistically significant (Fig. 1). However, no difference was observed in their frequent use of long sleeve clothing (aPOR 1.17; 95% CI 0.37–3.71; *P* = 0.78). We found similar results in sensitivity analysis when melanoma survivors who also had a history of another cancer were excluded (Fig. S2).

For healthcare access, we found similar patterns of health insurance coverage and routine place to go for healthcare between melanoma survivors and adults with no cancer history. Older age (aPOR 1.07; 95% CI 1.03–1.11; *P* < 0.001) was associated with higher prevalence of being covered by health insurance, while low-income (aPOR 0.16; 95% CI 0.02–1.47; *P* = 0.1) appeared to be somewhat associated with lower insurance coverage (Table 2). Similar results were noted in the sensitivity analysis after excluding melanoma survivors with a prior history of another cancer (Table S1).

**Table 2:** Demographic characteristics distinguishing with health care access from without, never smokers and former smokers from current smokers, among melanoma survivors aged ≥ 20 years, NHANES

	Unadjusted POR (95% CI)	*P* value	Adjusted* POR (95% CI)	*P* value
Covered by health insurance				
Age	1.06 (1.03, 1.08)	< 0.001	1.07 (1.03, 1.11)	< 0.001
Gender				
Female	Referent		Referent	
Male	0.95 (0.18, 5.08)	0.95	0.49 (0.05, 4.34)	0.51
Education level				
College degree or higher	Referent		Referent	
High school or less than high school	0.37 (0.06, 2.14)	0.26	0.48 (0.06, 3.61)	0.46
Poverty income ratio				
Middle- or high-income (> 1.3)	Referent		Referent	
Low-income (≤ 1.3)	0.16 (0.03, 0.83)	0.03	0.16 (0.02, 1.47)	0.10
Routine place to go for healthcare				
Age	1.05 (0.99, 1.11)	0.08	1.05 (0.98, 1.13)	0.18
Gender				
Female	Referent		Referent	
Male	1.43 (0.30, 6.85)	0.65	0.95 (0.11, 8.25)	0.96
Education level				
College degree or higher	Referent		Referent	
High school or less than high school	0.78 (0.19, 3.30)	0.73	0.60 (0.12, 2.98)	0.52
Poverty income ratio				
Middle- or high-income (> 1.3)	Referent		Referent	
Low-income (≤ 1.3)	2.70 (0.30, 24.42)	0.37	3.25 (0.27, 39.26)	0.34
Smoking status (former vs. never)				
Age	1.03 (1.00, 1.06)	0.03	1.02 (0.99, 1.06)	0.19
Gender				
Female	Referent		Referent	
Male	3.55 (1.72, 7.31)	< 0.001	3.78 (1.75, 8.16)	< 0.001
Education level				
College degree or higher	Referent		Referent	
High school or less than high school	2.10 (0.95, 4.65)	0.07	2.59 (1.07, 6.30)	0.04
Poverty income ratio				
Middle- or high-income (> 1.3)	Referent		Referent	
Low-income (≤ 1.3)	1.28 (0.52, 3.12)	0.20	1.15 (0.40, 3.35)	0.79
Smoking status (current vs. never)				
Age	0.99 (0.96, 1.02)	0.34	0.97 (0.94, 1.01)	0.12
Gender				
Female	Referent		Referent	
Male	2.04 (0.81, 5.16)	0.13	2.91 (1.05, 8.06)	0.04
Education level				
College degree or higher	Referent		Referent	
High school or less than high school	3.48 (1.38, 8.81)	0.008	4.12 (1.65, 10.29)	0.002
Poverty income ratio				
Middle- or high-income (> 1.3)	Referent		Referent	
Low-income (≤ 1.3)	1.84 (0.73, 4.63)	0.40	1.34 (0.47, 3.86)	0.59

*Adjusted POR estimates were adjusted for age, gender, education level, and poverty income ratio

Smoking status did not differ between melanoma survivors and individuals with no cancer history (Fig. 1). Among melanoma survivors, we found males (aPOR 2.91; 95% CI 1.05–8.06; *P* = 0.04) and those with no college degree (aPOR 4.12; 95% CI 1.65–10.29; *P* = 0.002) more likely to be current (vs. never) smokers (Table 2). Similar findings were observed in the sensitivity analysis when excluding melanoma survivors with a history of another cancer (Table S1).

## Discussion

Utilizing nationally representative data, only one-third of melanoma survivors used multiple sun protection methods, slightly more than adults without cancer. Melanoma survivors in NHANES had high levels of health insurance coverage and routine place to go for healthcare that did not differ from adults without a history of cancer. While smoking rates were similar, 16% of melanoma survivors were current smokers. These findings can inform targeted programs to improve melanoma survivorship, focusing on sun protection and smoking cessation.

While melanoma survivors were somewhat more likely to practice multimodal sun protection behaviors than adults without a cancer history, both groups had relatively low use of sun protection, including multiple practices; below 50%, with long sleeves showing the lowest adherence. Our findings revealed lower use of sun protection behaviors compared to prior studies conducted in different populations or during different time periods [34, 36, 49, 50]. Frequent engagement in sun protective practices may help reduce subsequent melanoma risk [51, 52]. Despite efforts in the US to educate the public, like those by the NCCN [9], American Academy of Dermatology [53], and American Cancer Society [54], many dismiss the messages [49], highlighting the challenge of motivating melanoma survivors. In contrast, in Australia, where skin cancer mass media campaigns have been in existence for a longer span of time, there has been a significant attitudinal shift, which has led to an increase in sunscreen use and protective clothing and a decrease in sunburn occurrences [55]. Several studies also showed public health programs designed to promote sun-protective behaviors seem to be gaining traction among adolescents and young adults in the US [45, 56–58].

For healthcare access, we found that only 2.1% of melanoma survivors lacked a routine healthcare provider, which is consistent with 2.2% from the National Institutes of Health “All of Us” Research Program [59]. Additionally, melanoma survivors appeared to have similar health insurance and routine place to go for care compared to adults without cancer history, consistent with other US studies on cancer survivors [60, 61]. A limitation of this study is that melanoma survivors without healthcare access who were diagnosed at an advanced stage and have since died would not be included. Thus, our findings might underestimate the magnitude of healthcare access disparities between participants with a history of melanoma and those with no cancer history. In addition, we found that melanoma survivors who were younger and low-income were somewhat less likely to be covered by health insurance, not surprising because Medicare, the federal health insurance program, is available mostly to US citizens aged 65 years or above [62]. To make affordable health insurance available to more people, the Affordable Care Act (ACA) allowed states, starting from January 1, 2014, to expand Medicaid coverage to a broad group of low-income people, subject to residency requirements [63]. Future work should continue to focus on developing targeted interventions to address these multifaceted barriers and promote equitable healthcare access for young and low-income populations.

Because of the absence of specific recommendations regarding smoking cessation for cutaneous melanoma survivors from US institutions, our study, like previous studies [29, 33, 64], revealed a notable 16% prevalence of current smoking among melanoma survivors. A cross-sectional analysis of the 2017 and 2020 US National Health Interview Survey (NHIS) similarly revealed that among melanoma survivors, the percentages of current smokers were 15% and 13%, respectively [29], suggesting that smoking status among melanoma survivors remained relatively consistent over the past few years. In agreement with previous findings among cancer survivors in general, we observed that melanoma survivors who were male or had an education level of high school or less were more likely to be current smokers [65, 66]. Overall, our findings suggest the underlying need for awareness and more uniform guidelines regarding smoking cessation, and developing smoking cessation programs customized to target gender-specific strategies and accessible to those with lower levels of education.

Behavioral changes remain key to improving melanoma survivorship, regardless of the presence of hereditary genetic alterations [67–71]. Around 7–15% of melanoma cases occur in individuals with a family history, often due to shared sun exposure rather than the inheritance of a single genetic mutation like *CDKN2A* [67, 68]. While genetic predispositions, such as *CDKN2A* mutations can increase melanoma risk, early diagnosis significantly improves outcomes [69, 70]. Notably, *CDKN2A* mutations are also linked to a higher risk of pancreatic cancer [72, 73], particularly among smokers [71], underscoring the importance of smoking cessation. Promising findings from the US Smoking Treatment and Recovery (STAR) program demonstrate the feasibility of tailored interventions in the oncology setting, with a majority of participants attempting to quit smoking and many utilizing cessation aids [74]. Besides smoking cessation program for all cancer survivors, educational programs in the US have focused on improving sun protection and skin self-examination among melanoma survivors, with web-based tools playing a growing role [74–78]. Recent studies show these interventions enhance intentions for sun protection and self-examinations [74, 75]. Additionally, involving partners in self-examination training improves outcomes compared to training patients alone [77, 78].

There were several limitations for this study. First, this study used cross-sectional, self-reported data, although such measures have been validated for assessing sun protection behaviors [79], healthcare access [80, 81], and smoking status [82–84]. Second, pooling data from multiple years may limit the generalizability of findings to a specific year, with much of the data being up to 10 years old, potentially affecting its generalizability for the current US population. Third, the survey did not identify which specific type of melanoma the patient had. Lastly, survey data on sun protection behaviors was age restricted to individuals 20–59 years old, limiting our ability to assess sun protection behaviors among melanoma survivors outside this age range.

## Conclusion

While we did not find many differences in sun protection, healthcare access, or smoking between melanoma survivors and those without cancer history, our study underscores the importance of increased sun protection and smoking cessation among melanoma survivors. Policymakers and healthcare providers should prioritize reinforcing sun protection habits and integrating smoking cessation support into survivorship guidelines, especially targeting males and individuals with lower education levels. Additionally, ensuring affordable health insurance options, particularly for young and low-income cancer survivors, remains crucial.

## Supplementary Information

Below is the link to the electronic supplementary material.

Supplementary file1 (DOCX 508 KB)

## Data Availability

The datasets generated during and/or analyzed during the current study are available via the National Center for Health Statistics (NCHS) website at https://wwwn.cdc.gov/nchs/nhanes/Default.aspx.
