# Supplementary material for: Sun protection behaviors, healthcare access, and smoking among melanoma survivors
Source: J Cancer Surviv. 2024 Dec 9;20(3):1264–73. doi: 10.1007/s11764-024-01727-8 (PMC13144187; doi:10.1007/s11764-024-01727-8)
Supplement: Supplementary file 1 — Supplementary file1 (DOCX 508 KB) [file 11764_2024_1727_MOESM1_ESM.docx]

**Supplementary Materials**

**Figure S1** Summary of selection of study participants and analytic populations. National Health and Nutrition Examination Survey (NHANES)


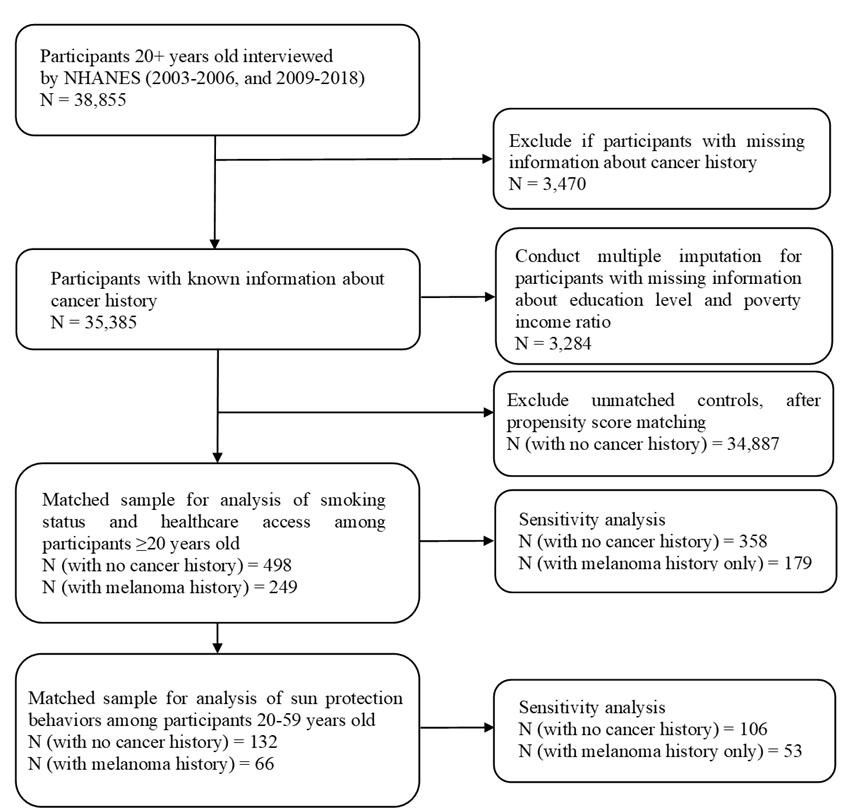


**Figure S2** Unadjusted and adjusted^*^ prevalence odds ratio (POR) and 95% confidence interval (CI) of health care access, smoking status and sun protection behaviors among melanoma survivors^#^ and matched adults with no cancer history, NHANES


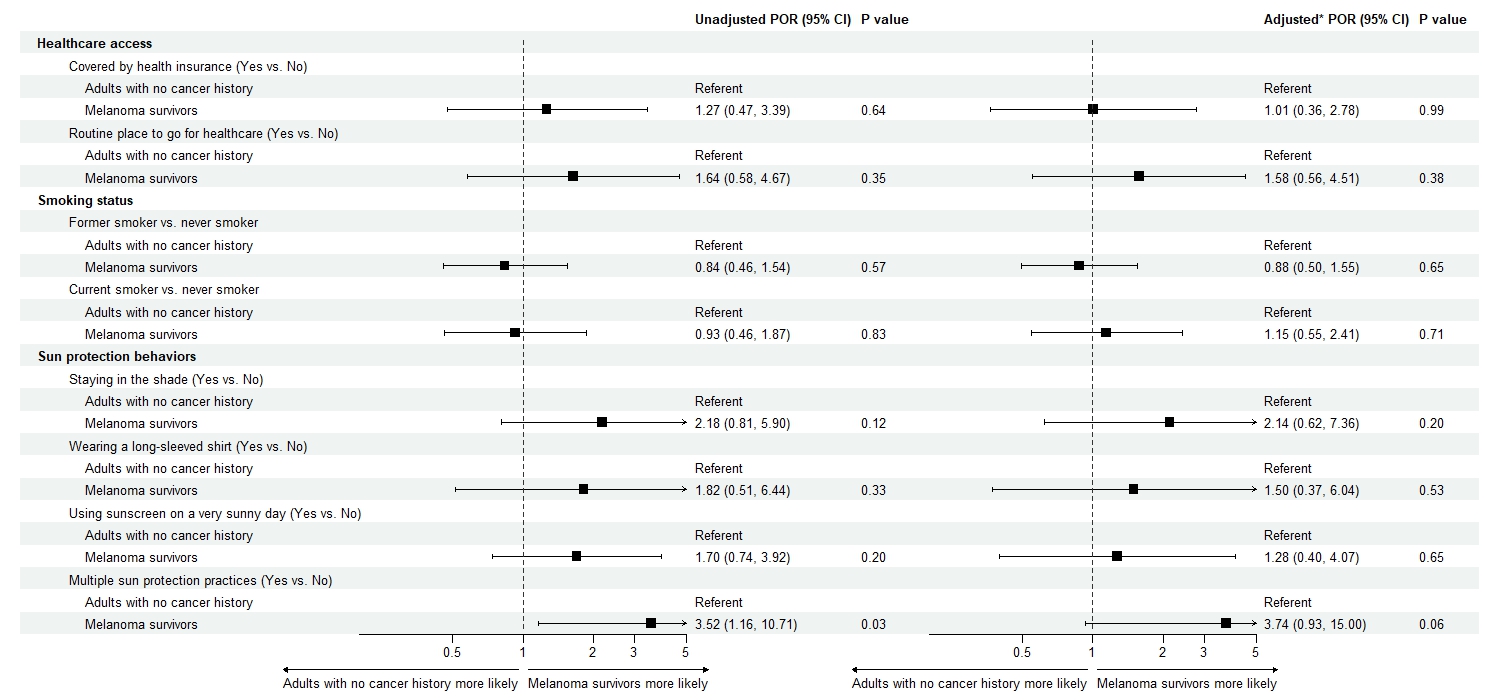


Those who reported no cancer history were used as the reference group.

^*^Adjusted POR estimates were adjusted for age, gender, race/ethnicity, education level, and poverty income ratio.

^#^For the outcome healthcare access and smoking status, excludes melanoma survivors with a history of another cancer (N=70) and their matching controls (N=140);

for the outcome sun protection behaviors, excludes melanoma survivors with a history of another cancer (N=13) and their matching controls (N=26).

**Table S1** Demographic characteristics distinguishing with health care access from without, never smokers and former smokers from current smokers, among melanoma survivors aged ≥20 years, NHANES

|  | **Unadjusted POR (95% CI)** | **P value** | **Adjusted**^*^ **POR (95% CI)** | **P value** |
| --- | --- | --- | --- | --- |
| **Covered by health insurance** |  |  |  |  |
| **Age** | 1.04 (1.02, 1.06) | <0.001 | 1.07 (1.02, 1.12) | 0.007 |
| **Gender** |  |  |  |  |
| Female | Referent |  | Referent |  |
| Male | 0.69 (0.11, 4.48) | 0.69 | 0.35 (0.02, 5.30) | 0.43 |
| **Education level** |  |  |  |  |
| College degree or higher | Referent |  | Referent |  |
| High school or less than high school | 0.50 (0.07, 3.43) | 0.47 | 0.57 (0.06, 5.37) | 0.61 |
| **Poverty income ratio** |  |  |  |  |
| Middle- or high- income (>1.3) | Referent |  | Referent |  |
| Low-income (≤1.3) | 0.22 (0.04, 1.33) | 0.09 | 0.11 (0.01, 1.90) | 0.13 |
| **Routine place to go for healthcare** |  |  |  |  |
| **Age** | 1.04 (0.98, 1.10) | 0.19 | 1.04 (0.96, 1.13) | 0.34 |
| **Gender** |  |  |  |  |
| Female | Referent |  | Referent |  |
| Male | 1.34 (0.26, 6.96) | 0.72 | 0.96 (0.10, 9.61) | 0.97 |
| **Education level** |  |  |  |  |
| College degree or higher | Referent |  | Referent |  |
| High school or less than high school | 0.84 (0.19, 3.66) | 0.81 | 0.66 (0.13, 3.36) | 0.61 |
| **Poverty income ratio** |  |  |  |  |
| Middle- or high- income (>1.3) | Referent |  | Referent |  |
| Low-income (≤1.3) | 2.72 (0.28, 26.21) | 0.37 | 2.23 (0.16, 31.70) | 0.54 |
| **Smoking status (former vs. never)** |  |  |  |  |
| **Age** | 1.04 (1.00, 1.07) | 0.03 | 1.02 (0.99, 1.06) | 0.16 |
| **Gender** |  |  |  |  |
| Female | Referent |  | Referent |  |
| Male | 2.67 (1.17, 6.13) | 0.02 | 2.71 (1.13, 6.52) | 0.03 |
| **Education level** |  |  |  |  |
| College degree or higher | Referent |  | Referent |  |
| High school or less than high school | 2.58 (0.97, 6.87) | 0.06 | 2.84 (1.00, 8.03) | 0.05 |
| **Poverty income ratio** |  |  |  |  |
| Middle- or high-income (>1.3) | Referent |  | Referent |  |
| Low-income (≤1.3) | 1.43 (0.51, 4.00) | 0.50 | 1.05 (0.32, 3.45) | 0.93 |
| **Smoking status (current vs. never)** |  |  |  |  |
| **Age** | 1.00 (0.97, 1.03) | 0.87 | 0.98 (0.95, 1.02) | 0.40 |
| **Gender** |  |  |  |  |
| Female | Referent |  | Referent |  |
| Male | 2.29 (0.84, 6.24) | 0.11 | 2.88 (0.95, 8.70) | 0.06 |
| **Education level** |  |  |  |  |
| College degree or higher | Referent |  | Referent |  |
| High school or less than high school | 2.91 (1.10, 7.74) | 0.03 | 3.62 (1.36, 9.62) | 0.01 |
| **Poverty income ratio** |  |  |  |  |
| Middle- or high- income (>1.3) | Referent |  | Referent |  |
| Low-income (≤1.3) | 0.81 (0.20, 3.23) | 0.77 | 0.70 (0.14, 3.51) | 0.67 |

^*^Adjusted POR estimates were adjusted for age, gender, education level, and poverty income ratio.

^#^Excludes melanoma survivors with a history of another cancer (N=70).
